# Chest‐lead ST‐J amplitudes using arm electrodes as reference instead of the Wilson central terminal in smartphone ECG applications: Influence on ST‐elevation myocardial infarction criteria fulfillment

**DOI:** 10.1111/anec.12549

**Published:** 2018-05-07

**Authors:** Thomas Lindow, Henrik Engblom, Ardavan Khoshnood, Ulf Ekelund, Marcus Carlsson, Olle Pahlm

**Affiliations:** ^1^ Department of Clinical Physiology Växjö Central Hospital Växjö Sweden; ^2^ Department of Clinical Sciences Lund Clinical Physiology Skane University Hospital Lund University Lund Sweden; ^3^ Department of Clinical Sciences Lund Emergency Medicine Skane University Hospital Lund University Lund Sweden

**Keywords:** CL leads, CR leads, smartphone 12‐lead ECG, ST‐elevation myocardial infarction criteria, Wilson central terminal

## Abstract

**Background:**

“Smartphone 12‐lead ECG” for the assessment of acute myocardial ischemia has recently been introduced. In the smartphone 12‐lead ECG either the right or the left arm can be used as reference for the chest electrodes instead of the Wilson central terminal. These leads are labeled “CR leads” or “CL leads.” We aimed to compare chest‐lead ST‐J amplitudes, using either CR or CL leads, to those present in the conventional 12‐lead ECG, and to determine sensitivity and specificity for the diagnosis of STEMI for CR and CL leads.

**Methods:**

Five hundred patients (74 patients with ST elevation myocardial infarction (STEMI), 66 patients with nonischemic ST deviation and 360 controls) were included. Smartphone 12‐lead ECG chest‐lead ST‐J amplitudes were calculated for both CR and CL leads.

**Results:**

ST‐J amplitudes were 9.1 ± 29 μV larger for CR leads and 7.7 ± 42 μV larger for CL leads than for conventional chest leads (V leads). Sensitivity and specificity were 94% and 95% for CR leads and 81% and 97% for CL leads when fulfillment of STEMI criteria in V leads was used as reference. In ischemic patients who met STEMI criteria in V leads, but not in limb leads, STEMI criteria were met with CR or CL leads in 91%.

**Conclusion:**

By the use of CR or CL leads, smartphone 12‐lead ECG results in slightly lower sensitivity in STEMI detection. Therefore, the adjustment of STEMI criteria may be needed before application in clinical practice.

## 1 BACKGROUND

Smartphone technology in cardiology is developing fast and has great potential because of its widespread availability (Martínez‐Pérez, de la Torre‐Díez, López‐Coronado, & Herreros‐González, 2013). For example, smartphone‐based one‐lead recording of electrocardiograms (ECG) has been validated for the diagnosis of atrial fibrillation (Lau et al., 2013). If smartphone ECG with recording capability for all 12 leads would become a reliable substitute for the conventional 12‐lead ECG, there is a potential for very early detection of ST‐elevation myocardial infarction (STEMI) upon symptom onset, even at the patient's home. Also, the smartphone could substitute for an ECG machine in healthcare settings where ambulance infrastructure is underdeveloped or ECG machines are scarce.

**Figure 1:**
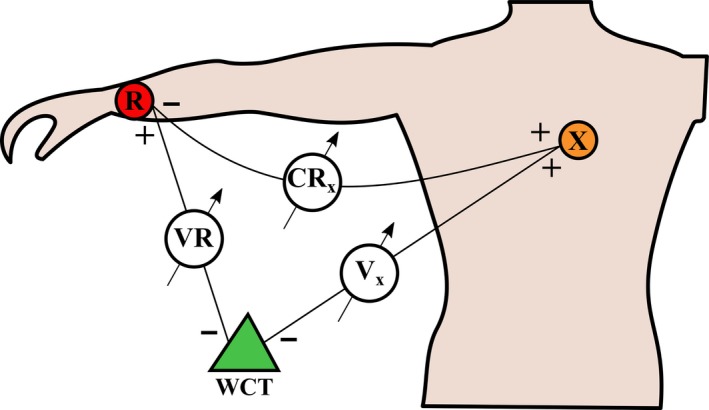
Electrode arrangement for recording leads Vx and CRx. R denotes the right arm electrode, and x denotes one of the six chest electrodes (1–6). WCT denotes the Wilson Central Terminal. The potentials indicated in the voltmeter symbols (Vx, CRx, and VR) obey Kirchoff′s second law, that is, CRx = Vx – VR = Vx − $\left(\frac{2}{3}\right)$ aVR. The same principles apply to all chest leads (V1–V6, CR1–CR6). If the left arm is used as reference, VL and aVL will replace VR and aVR in the formula

Smartphone technology for assessment of acute myocardial ischemia by the generation of “smartphone 12‐lead ECG” has recently been introduced (Muhlestein et al., 2015). In that application the three limb leads, I, II, and III, are recorded by placing adhesive electrode tabs on the left arm (L), right arm (R), and on the left leg (F), similar to the procedure for recording the conventional 12‐lead ECG. One difference, however, is that the leads are recorded sequentially, not simultaneously. Another difference is the reference electrode for the chest leads. When recording the conventional 12‐lead ECG, the chest leads are created by subtracting the potential at the so‐called Wilson central terminal (WCT) from the potential at each chest electrode (C1, …, C6). The WCT is the average potential of the three limb potentials, R, L, and F (Gargiulo, 2015; Kligfield et al., 2007). In the smartphone 12‐lead ECG application, the right or the left arm is used as reference instead of the WCT (Baquero, Banchs, Ahmed, Naccarelli, & Luck, 2015; Muhlestein et al., 2015). The resulting PQRST waveforms of CR and CL leads are not identical to those of the corresponding leads recorded with the WCT reference. It is not known how this affects the accuracy of diagnosis of STEMI. In the ongoing ST LEUIS trial, smartphone 12‐lead ECG is compared to conventional 12‐lead ECG by sequentially performing simultaneous recordings of a conventional chest lead (V lead) and the corresponding CR or CL lead (Barbagelata et al., 2017). However, the comparison of ST‐J amplitudes in V leads to those in CR or CL leads does not require such a recording procedure. The ECG waveforms and thus the ST‐J amplitudes, as they would appear in the smartphone application, can be calculated from the V leads and the relevant augmented lead (aVR or aVL) in the conventional 12‐lead ECG (Figure 1).

The purpose of this study was to compare the chest‐lead ST‐J amplitudes, using either the right or left arm electrode as reference, to those in the conventional 12‐lead ECG. Also, we aimed to determine sensitivity and specificity for the diagnosis of STEMI for smartphone 12‐lead ECG based on these reference electrodes, and to compare them to those obtained with the conventional 12‐lead ECG.

## 2 METHODS

A total of 500 ECGs from patients from three different study populations were included in this study. Seventy‐four patients with STEMI (37 with a culprit lesion in the left anterior descending artery (LAD), 32 in the right coronary artery (RCA) and 5 in the left circumflex artery (LCx)) were recruited from the SOCCER study (Khoshnood et al., 2016). The SOCCER study included 95 patients referred for primary percutaneous coronary intervention (PCI) who had been randomized to either standard oxygen therapy or no supplemental oxygen. Patients with an ECG without significant ST elevation in two contiguous leads (*n* = 19) or a technically deficient ECG (*n* = 2), were excluded from this study. Fifty‐one patients had significant ST elevation in two contiguous chest leads and thus met STEMI criteria in V leads (LAD *n* = 37, RCA *n* = 10, LCx *n* = 4). Among these patients, 33 patients met STEMI criteria in V leads, but not in limb leads. Twenty‐three patients had significant ST elevation in two contiguous limb leads, but did not meet STEMI criteria in V leads.

Sixty‐six patients with nonischemic ST deviation due to pericarditis, (*n* = 26), early repolarization syndrome (ERS) (*n* = 14) or left ventricular hypertrophy (LVH) (*n* = 26) were included from another study (Akil et al., 2013). These ECGs were retrieved from a clinical ECG database and identified by a search in the interpretive statements. Confirmation of the clinical diagnosis was performed by reviewing patient records. In addition, 360 patients without ongoing myocardial ischemia (Lindow, Olson, Swenne, Man, & Pahlm, 2017) were included and served as controls. This dataset consisted of 30 ECGs for each gender and each age decade (30–39, 40–49,…, 80–89). All ECGs had been recorded either before a planned exercise test or myocardial perfusion imaging, before Holter monitoring or as a screening ECG before noncardiac surgery. Only patients with a very low likelihood of ongoing transmural ischemia were thus included. ST‐J amplitudes in all 12 leads were measured at the J‐point, that is, the end of QRS/beginning of ST segment, and the same point in time was used for all 12 leads. The amplitude level immediately before the beginning of QRS was designated as the zero level for each lead (Rautaharju, Surawicz, & Gettes, 2009).

ST‐J amplitudes for smartphone chest leads were calculated when either the right arm (R) or the left arm (L) electrode potential was used as reference. The ST‐J amplitude in a smartphone‐ECG chest lead “x” was calculated as follows:

$$\text{R as reference}:\;\text{CRx}\;=\;\text{Vx}-\frac{2\times \text{aVR}}{3}$$

$$\text{L as reference}:\;\text{CLx}\;=\;\text{Vx}-\frac{2\times \text{aVL}}{3}$$

STEMI criteria (ST‐J elevation ≥0.1 mV in all leads except V2 and V3 (≥0.15 mV for women, ≥0.2 mV men ≥40 years, 0.25 mV men <40 years) (Thygesen et al., 2012) were applied on V leads as well as CR and CL leads in all patients.

Necessary ethical approvals by the ethical review board were obtained for the studies from which the ECGs were included. Written informed consent was either obtained (Khoshnood et al., 2016) or waived by the ethical boards (Akil et al., 2013; Lindow et al., 2017).

### 2.1 Statistical analysis

Descriptive statistics are presented as mean ± standard deviation. Student's *t* test was used for comparison of mean ST‐J amplitudes between CR/CL leads and conventional 12‐lead ECG leads (V leads). Pearson correlation test was used to assess correlation between ST‐J amplitudes in conventional and smartphone ECG. When calculating sensitivity and specificity, fulfillment of STEMI criteria in V leads was considered reference standard. For example, when STEMI‐criteria were fulfilled in both V leads and CR leads, the test result was considered true positive, and if they were met in CR leads but not in V leads, the result was considered false positive. Sensitivity and specificity are described with 95% confidence intervals. A *p*‐value of <.05 was considered statistically significant.

## 3 RESULTS

**Figure 2:**
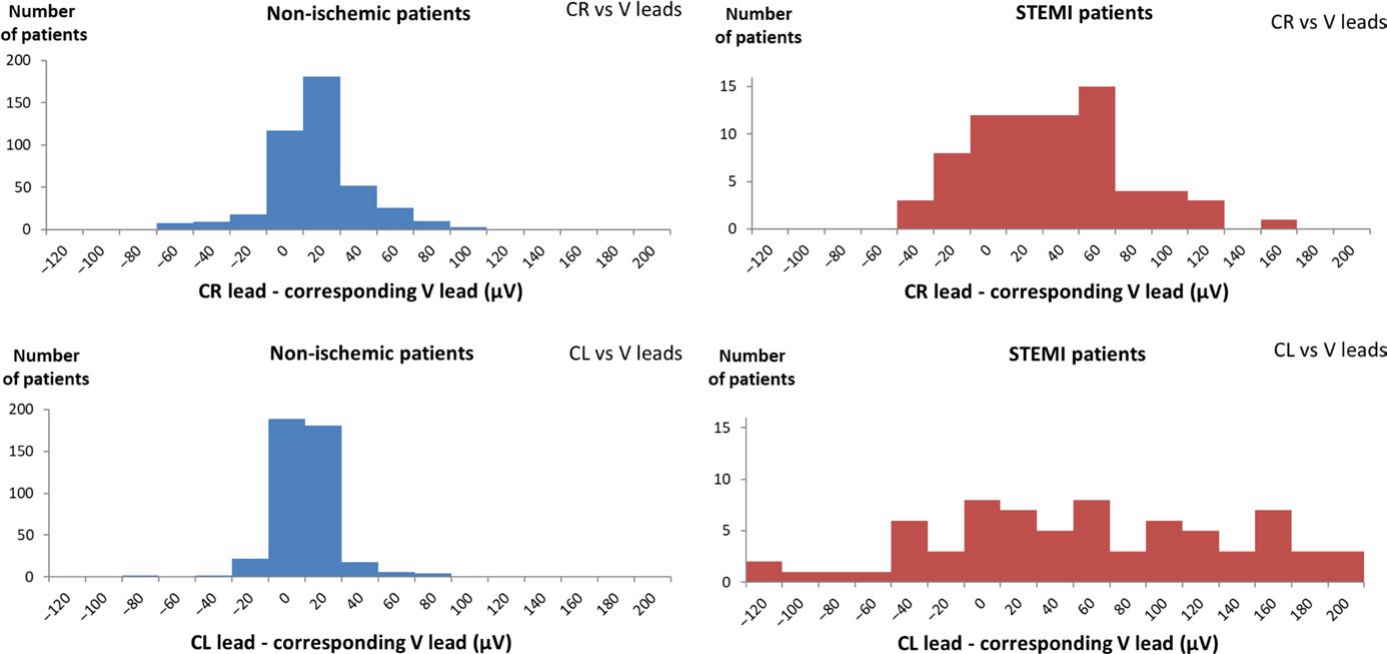
Differences between CR/CL amplitudes and the corresponding V amplitudes described with histograms for nonischemic patients (*n* = 426, blue bars) and STEMI patients (*n* = 74, red bars). The bars represent number of patients with a difference in ST‐J‐amplitude between CR/CL and V lead of <−120 μV, −100 to −120 μV, …, 180–200 μV, >200 μV. Upper panel: CR leads vs. V leads. Lower panel: CL leads vs. V leads. In nonischemic patients, the difference in ST‐J amplitude compared to V leads is larger in CR leads than in CL leads, whereas the opposite is found in STEMI patients

ST‐J amplitudes for the entire study population (*n* = 500) were 9.1 ± 29 μV larger (*p* < .001) using the right arm as reference (CR leads) instead of the WCT and 7.7 ± 42 μV larger (*p* < .001) using the left arm as reference (CL leads). Mean ST‐J amplitudes for all leads are presented in Table 1. In STEMI patients, ST‐J amplitudes were 25.0 ± 41.8 μV larger for CR leads compared to V leads and 52 ± 90.5 μV larger for CL leads. The difference in ST‐J amplitudes between CR/CL leads and V leads are described in Figure 2. In nonischemic patients, the difference in ST‐J amplitude compared to V leads was found to be slightly larger in CR leads than in CL leads, whereas the opposite was found in STEMI patients.

**Table 1:** Mean ST‐J amplitudes (μV) in V leads, CR leads, and CL leads

	V1	CR1	CL1	V2	CR2	CL2
All patients	25 (57)	34 (49)	32 (63)	64 (146)	18 (149)	72 (126)
Controls	26 (26)	31 (28)^a^	25 (29)	58 (52)	63 (337)^a^	57 (291)^a^
STEMI	15 (123)	40 (102)^a^	67 (117)^a^	76 (349)	101 (337)	128 (291)^a^
Nonischemic ST deviation	29 (66)	42 (48)	33 (93)	86 (105)	21 (111)	91 (91)

	V3	CR3	CL3	V4	CR4	CL4
All patients	58 (144)	68 (154)	66 (132)	32 (112)	41 (129)	40 (107)
Controls	43 (51)	48 (61)^a^	42 (49)	20 (43)	25 (54)^a^	19 (41)
STEMI	125 (325)	150 (319)^a^	178 (282)^a^	102 (208)	127 (212)^a^	154 (184)^a^
Nonischemic ST deviation	66 (142)	79 (192)	71 (120)	21 (177)	35 (229)	26 (154)

	V5	CR5	CL5	V6	CR6	CL6
All patients	12 (82)	21 (107)	20 (86)	7 (61)	18 (88)	20 (86)
Controls	4 (30)	9 (43)^a^	3 (29)	3 (23)	9 (36)^a^	3 (29)
STEMI	62 (114)	87 (141)	114 (132)	36 (93)	61 (129)^a^	114 (132)^a^
Nonischemic ST deviation	1 (171)	14 (222)	5 (86)	7 (61)	20 (88)	5 (86)

a *p*‐value <.05. The *p*‐value refers to the comparison of mean ST‐J amplitudes in the CR or CL lead and the corresponding V lead.

**Figure 3:**
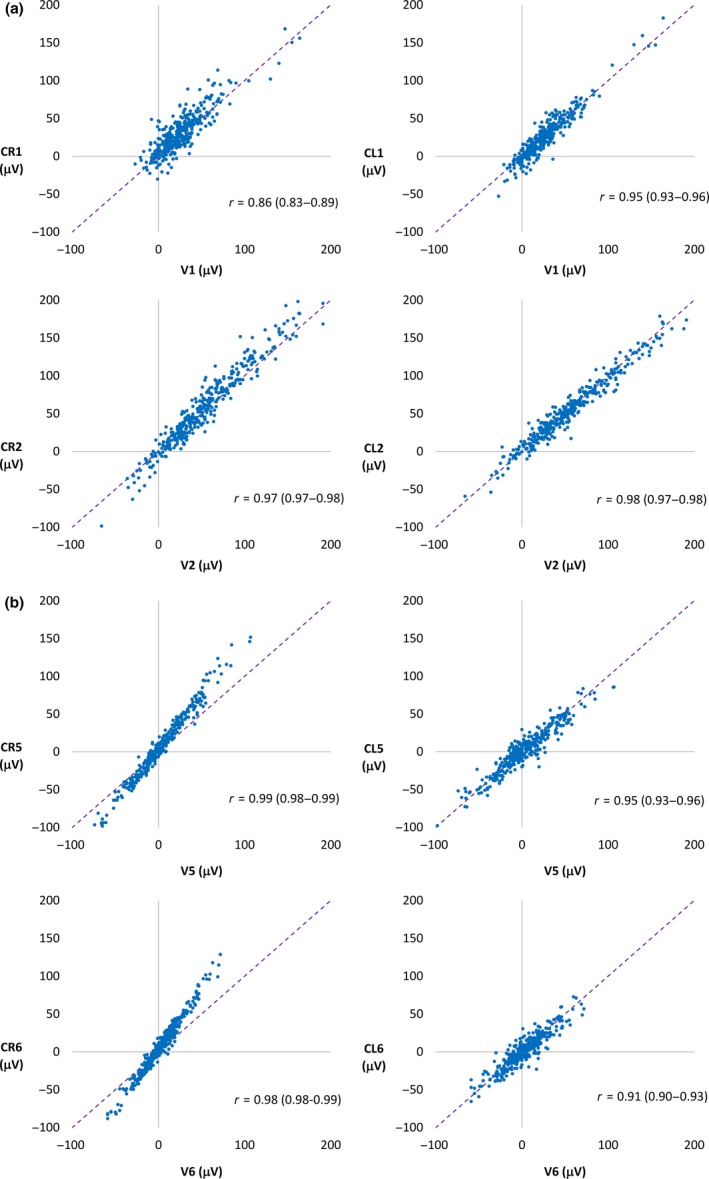
Nonischemic controls. Scatter plots of ST‐J amplitudes (μV) with ST‐J amplitudes in V leads on the *x* axis and in CR (left panel) and CL (right panel) leads on the *y* axis The purple dashed line represents the identity line. *R*‐values are presented with 95% confidence intervals. (a) Electrode positions C1 and C2 on the chest. (b) Electrode positions C5 and C6 on the chest. CR lead amplitudes deviate from the identity line, to a greater extent, in lateral than in septal leads

**Figure 4:**
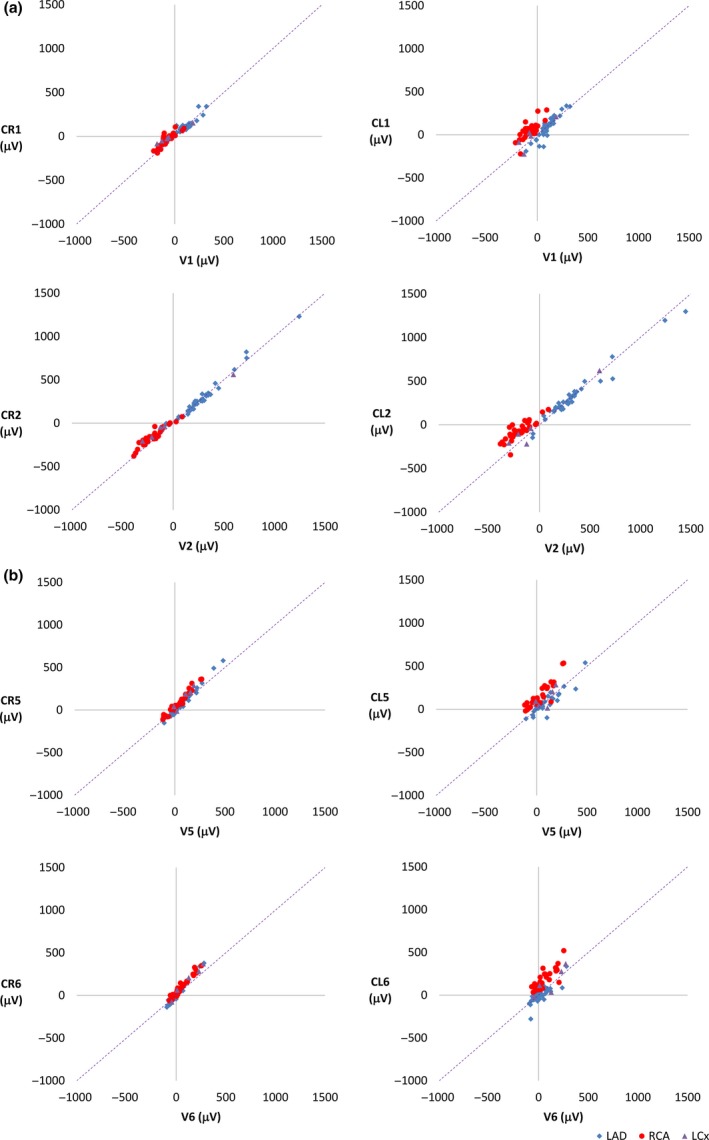
Patients with STEMI. Scatter plots of ST‐J amplitudes (μV) with ST‐J amplitudes in V leads on the *x* axis and in CR and CL leads on the *y* axis, CR leads in the left panel and CL leads in the right panel. The purple dashed line represents the identity line. LAD patients are represented as blue diamonds, RCA patients as red circles and LCx patients as green triangles. (a) Electrode positions C1 and C2 on the chest. (b) Electrode positions C5 and C6 on the chest. To a greater extent than for CR leads, CL lead amplitudes deviate from the identity line

In controls, correlation with lead V1 was higher for CL1 than for CR1 (Figure 3a), whereas the reverse was observed for the lateral leads (CR5, CR6, CL5, CL6) (Figure 3b). For leads V2–V4, correlations were similar for CL and CR leads. CR lead amplitudes deviated from the identity line in chest leads 2–6 to a larger extent than for CL lead amplitudes (Figure 3). In STEMI patients, the opposite was found, with greater deviation from the identity line in CL leads (Figure 4).

For all patients, sensitivity and specificity were 94% (87–98) and 95% (93–97) for CR leads when fulfillment of STEMI criteria in the conventional 12‐lead ECG was used as reference standard; for CL leads sensitivity and specificity were 81% (71–88) and 97% (95–99). STEMI criteria were met in V leads in 51 STEMI patients. In 33 patients, STEMI criteria were met in V leads, but not in limb leads. Among these patients, STEMI criteria were met in 30 patients (91%) in both CR and CL leads. STEMI patients without significant ST elevation in two contiguous chest leads, that is, where STEMI criteria were not met in V leads, (22 patients with RCA culprit, 1 LCx), two patients had significant ST elevation in two contiguous chest leads using CR leads (9%) and nine using CL leads (39%).

In nonischemic patients, that is, controls and patients with nonischemic ST deviation, STEMI criteria in V leads on the conventional 12‐lead ECG were not met in 399 patients. Ninety‐five percent and 99% of these patients remained negative using CR leads and CL leads, respectively. In nonischemic controls, STEMI criteria were fulfilled in three patients using conventional 12‐lead ECG and in CR and CL leads in 15 and 2 patients, respectively.

With conventional 12‐lead ECG, STEMI criteria were falsely positive in V leads in 33 patients with nonischemic ST deviation (pericarditis *n* = 23, ERS *n* = 10, LVH *n* = 1). Ninety‐seven percent of these patients remained positive with CR leads and 71% with CL leads. Detailed information on sensitivity and specificity is presented in Table 2.

**Table 2:** Sensitivity and specificity regarding STEMI criteria fulfillment in chest leads using the conventional 12‐lead ECG as reference standard

**All patients (** ***n*** ** = 500)**			
	True positive	False negative	Sensitivity (%)
CR	83	5	94
CL	71	17	81
	True negative	False positive	Specificity (%)
CR	392	20	95
CL	401	11	97
**STEMI patients (** ***n*** ** = 74)**			
	True positive	False negative	Sensitivity (%)
CR	47	4	92
CL	46	5	90
	True negative	False positive	Specificity (%)
CR	21	2	91
CL	14	9	61
**No STEMI (controls and nonischemic ST deviation, ** ***n*** ** = 426)**			
	True positive	False negative	Sensitivity (%)
CR	36	1	97
CL	25	12	68
	True negative	False positive	Specificity (%)
CR	371	18	95
CL	387	2	99
**Patients with nonischemic ST deviation (** ***n*** ** = 66)**			
	True positive	False negative	Sensitivity (%)
CR	33	1	97
CL	24	10	71
	True negative	False positive	Specificity (%)
CR	26	6	81
CL	31	0	100

## 4 DISCUSSION

This study shows that replacement of the WCT by arm electrodes, in most patients, results in only small changes in ST‐J amplitudes. However, in patients with STEMI or other diagnoses which affect ST‐J amplitudes in leads aVR and/or aVL, changes in precordial‐lead ST‐J amplitudes may be substantial. This resulted in the changes in STEMI criteria fulfillment in some patients.

Before WCT became standard, both the left arm and the right arm electrodes were explored for use as reference for recording chest leads (Edwards & Vander Veer, 1938; Kossmann, 1985; Wolferth & Wood, 1932). In Scandinavia, CR leads were used until the 1970s, especially at departments where exercise ECG was performed, since ECG‐waveform morphologies in CR leads differ very little from those in chest‐head (CH) leads, which were used in exercise testing (Åstrand et al., 1967; Holmgren & Strandell, 1961; Jorfeldt, 1975). Since physicians are familiar with V leads, and diagnostic criteria have been developed for them, any difference in ECG patterns or amplitudes introduced by recording CR or CL leads may have important clinical implications. Åstrand et al. (1967) compared V leads to CR leads, and found higher amplitudes in lateral CR leads than in V leads and recommended a change in the ST‐elevation criteria for lateral chest leads. This is in agreement with the findings in our study, with larger difference in amplitudes between CR leads and V leads in lateral leads compared to septal leads (Figure 3).

**Figure 5:**
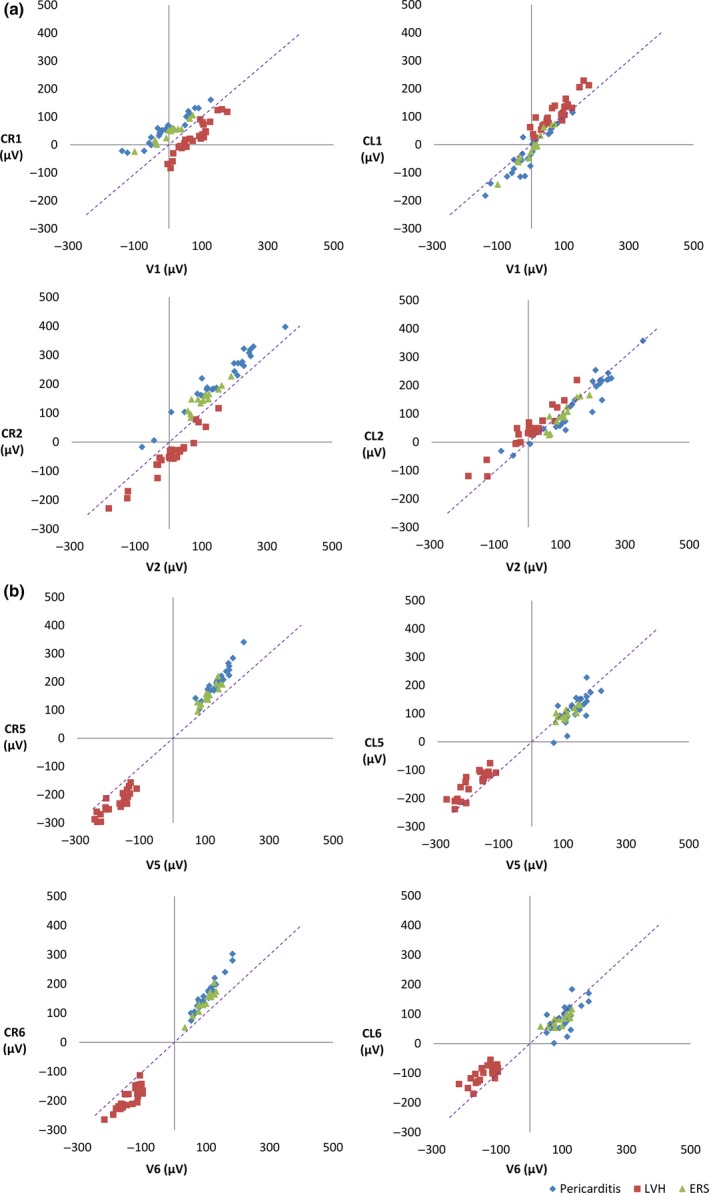
Patients with nonischemic ST deviation. Scatter plots of ST‐J amplitudes (μV) with ST‐J amplitudes in V leads on the *x*‐axis and in CR and CL leads on the *y*‐axis, CR leads in the left panel and CL leads in the right panel. The purple dashed line represents the identity line. Patients with pericarditis are represented as blue diamonds, LVH patients as red squares and ERS patients as green triangles. (a) Electrode positions C1 and C2 on the chest. (b) Electrode positions C5 and C6 on the chest

There are situations where ST‐deviation patterns differ significantly between CR/CL leads and V leads. For example, in patients with ST elevation in aVR or aVL, CR‐ or CL‐lead ST amplitudes will be diminished compared to V‐lead ST amplitudes. In patients with proximal LAD occlusion, ST elevation in aVR or aVL may be present (Atar & Birnbaum, 2005; George, Arumugham, & Figueredo, 2010) and CR‐ or CL‐lead amplitudes will be diminished. On the other hand, if ST depression is present in aVR or aVL in patients with inferior STEMI, CR or CL leads could show a pattern of widespread ST elevation, and may emulate a pericarditis pattern. In the present study, this was the case for 9 of 23 of STEMI patients without significant ST elevation in the chest leads in the conventional ECG, when CL leads were used and for two patients when CR leads were used. STEMI patients with ST elevation in lead III often have ST depression in aVL (Perron, Lim, Pahlm‐Webb, Wagner, & Pahlm, 2007). In patients with pericarditis, chest lead amplitudes were instead diminished when CL leads were used (Figure 5), which could obscure the typical diagnostic pattern of widespread ST elevation in pericarditis (Wang, Asinger, & Marriott, 2003). It should be noted that even though specificity was high for both CR and CL leads (95% vs. 97%), the use of CR leads increased the number of false positive STEMI from 3 to 15 patients in nonischemic controls. In a population of patients with suspected acute coronary syndrome this increase appears acceptable. If, on the other hand, a 12‐lead smartphone ECG is used as a screening tool in patients with low likelihood of having acute coronary syndrome an increased number of false positive STEMI will have to be considered.

Several technical issues regarding 12‐lead ECG recording with a smartphone remain to be addressed. In conventional 12‐lead ECG, in modern electrocardiographs, simultaneous recording allows for simultaneous measurement of amplitudes in all leads (Paul Kligfield et al., 2007). In the smartphone‐ECG recording, the leads are sequentially recorded, which can make J point detection difficult. Since single‐lead measurements have been shown to underestimate, for example, QRS durations (Kligfield et al., 2007), the timing of the J point may differ from what would have been measured by simultaneous recording. Furthermore, conventional 12‐lead ECG recording is performed by medical staff with the patient in supine position. It is plausible that smartphone‐ECG recordings will be performed in an upright or semirecumbent position, in prehospital settings, for instance, at the patient's home. ECG changes due to an altered body position have been reported in ST‐monitoring (Adams & Drew, 1997). In 12‐lead ECG recording performed in supine and upright position, only small changes have been reported (Baevsky, Haber, Blank, & Smithline, 2007; Madias, 2006). Neither of these studies, however, were performed in STEMI patients.

In conventional 12‐lead ECG recording, lead misplacement is common (Rudiger, Hellermann, Mukherjee, Follath, & Turina, 2007), and ischemic patterns can be both missed and falsely introduced (Bond et al., 2012; Schijvenaars, Kors, van Herpen, Kornreich, & van Bemmel, 1997). Chest electrodes are often misplaced even when ECGs are recorded by experienced ECG technicians (Wenger & Kligfield, 1996). The risk of misplacement would most likely be increased when a new method is applied, especially in the hands of people without medical training. Although this matter is not covered in this article, we would recommend choosing either the right arm *or* the left arm as reference for the entire recording procedure—that is, not using different reference for different chest leads—as this would likely increase the risk of lead placement errors.

## 5 CONCLUSIONS

By the use of CR or CL leads, smartphone 12‐lead ECG results in slightly lower sensitivity in STEMI detection. Therefore, adjustment of STEMI criteria may be needed before application in clinical practice.

## CONFLICTS OF INTEREST

None (all authors).
